# Utility of the Milan System for Reporting Salivary Gland Cytopathology in Parotid Gland Masses: The Experience of Two Tertiary Centers

**DOI:** 10.7759/cureus.49259

**Published:** 2023-11-22

**Authors:** Saleh Alqaryan, Shmokh Alsalamah, Rafeef AlHajress, Latefa Alareek, Bushra Alharbi, Majed Albarrak, Mohammad Almayouf, Saleh Aldhahri, Mohammed Al essa, Khalid Al-Qahtani

**Affiliations:** 1 Department of Otolaryngology, Head and Neck Surgery, College of Medicine, King Saud University, Riyadh, SAU; 2 Division of Otorhinolaryngology-Head and Neck Surgery, Department of Surgery, King Abdulaziz Medical City Riyadh, Riyadh, SAU; 3 Department of Otolaryngology-Head and Neck Surgery, King Fahad Medical City, Riyadh, SAU

**Keywords:** cytopathology, salivary gland, malignancy, fine needle aspiration, parotid

## Abstract

Background

The application of fine needle aspiration (FNA) in parotid masses via the Milan System for Reporting Salivary Gland Cytopathology (MSRSGC) enhances the diagnosis of these lesions alongside radiological investigations.

Objectives

Our objective was to assess the risk of malignancy, sensitivity, specificity, and false positive and negative results for each category of the MSRSGC. Additionally, we assessed the level of agreement between the FNA results using MSRSGC and post-resection histopathological diagnosis.

Methods

We conducted a retrospective chart review of parotid gland masses that received FNA and postoperative pathological diagnosis at King Saud University Medical City and King Fahad Medical City between 2018 and 2022. We summarized the categorical variables using frequencies and percentages.

Results

A total of 172 cases met the inclusion criteria. Males encompassed 102 patients (59.3%) of the study sample, and 89 (51.7%) of parotid masses were on the left side. The risk of malignancy for the MSRSGC categories was 37.5% (Category I), 9.0% (II), 50.0% (III), 4.7% (IVa), 50.0% (IVb), 100.0% (V), and 71.0% (VI). FNA had an overall success rate of 81%. The sensitivity was 64% and specificity was 94% for non-neoplastic masses. For benign masses, the sensitivity was 91% and specificity was 66%; however, the sensitivity was 40% and specificity was 97% for malignant lesions. We found that the percentage of agreement between the FNA and final pathology was 80%.

Conclusion

FNA using MSRSGC is a valuable preoperative clinical tool. However, the low sensitivity rates based on the diagnosis of malignant lesions should alert clinicians not to be overly reliant on biopsy results and instead defer to definitive surgical management.

## Introduction

Salivary gland tumors represent a challenging and difficult subset of neoplasms within head and neck tumors [1,2]. Their complex nature and intricate histological characteristics contribute to their classification as rare entities, accounting for merely 3-6% of all reported cases [1,2]. The occurrence of these tumors varies based on location, with approximately 70-85% arising in the parotid gland, 10-15% in the submandibular gland, and 5-10% in the sublingual and minor salivary glands [1,3]. Notably, the majority of parotid tumors (80-85%) are benign, while around 50% of submandibular tumors and 10% of sublingual tumors are benign [1,3]. However, detecting lesions in the parotid gland can be complex due to the existence of more than 40 different types of growths, both benign and malignant. Additionally, the intraparotid lymph nodes may contain variants of metastatic tumors. The treatment of both benign and malignant tumors primarily involves surgical resection [4].

Fine needle aspiration (FNA), a technique known for its simplicity and cost-effectiveness, has been widely employed for initially diagnosing salivary gland lesions [5-7]. However, inconsistent reporting of FNA across institutions has led to confusion among pathologists and clinical challenges [5]. There is debate surrounding FNA’s necessity in preoperative decisions due to its low sensitivity and variations in technique and interpretation [6,7]. To address this issue and enhance diagnostic accuracy, the Milan System for Reporting Salivary Gland Cytopathology (MSRSGC) has been introduced as an evidence-based classification system [8,9]. This system offers a comprehensive 6-tier diagnostic structure that categorizes specimens and provides the risk of malignancy (ROM) for each category-nondiagnostic (I), non-neoplastic (II), atypia of unknown significance (AUS) (III), benign neoplasm (IV-A), salivary gland neoplasm of unknown malignant potential (IV-B), suspected malignant (V), and malignant (VI)-accompanied by recommended management guidelines [8].

The present study investigates the diagnostic accuracy of FNA utilizing the MSRSGC to precisely delineate the ROM for each diagnostic category. Furthermore, this study seeks to assess the degree of concordance between the FNA results using the MSRSGC and the subsequent histopathological diagnosis via post-surgical resection.

## Materials and methods

A retrospective cohort study was conducted at King Saud University Medical City and King Fahad Medical City. Inclusion criteria included both adult and pediatric patients who presented with parotid masses and had been operated on between the years 2018 and 2022 and required the presence of both preoperative fine needle biopsy results and postoperative final pathological findings. To ensure homogeneity and geographical relevance, patients who had their fine needle biopsy or surgical procedures outside the purview of King Saud University Medical City and King Fahad Medical City were excluded. Additionally, individuals diagnosed with salivary gland masses other than those localized within the parotid gland, as well as, patients who had undergone preoperative open biopsies or those who lacked preoperative diagnosis were excluded from this study. 

We pursued and successfully obtained ethical approval from the Institutional Review Board (IRB) at King Fahad Medical City Hospital with approval number H-01-R-012 on the 3rd of October 2022, log number 22-455. Subsequently, oral consent was taken from patients who presented with parotid gland masses and underwent FNA, followed by postoperative pathological diagnoses.

The study was a retrospective chart review, and we summarized the categorical variables using frequencies and percentages. We analyzed the distribution of sample traits by three different methods, as appropriate. For overall distribution into Milan categories, we used Chi-squared followed by estimating the marginal means with Tukey adjustment. We assessed the distribution of gender, Past Medical History (PMH)+/−, right/left side, and additional treatment (Rx+/−) by Milan category using Bayesian logistic regression. We assessed the possible effect of age, height, weight, and year on the Milan category by generalized linear models. We tested conditional associations between Milan categories and risks of non-neoplastic, benign, or malignant states with multinomial logistic regression. We also tested population sample covariates alongside the Milan category to assess the effects on the success rate of FNA versus pathology. We compared competing models using the Bayesian information criterion (BIC); we selected the lowest BIC model as the most parsimonious. We analyzed the confusion matrix of FNA versus pathology-based diagnosis with several classifier evaluation metrics appropriately modified for a multiclass sample. All the data were analyzed using SPSS version 23 (IBM Corp., Armonk, NY, USA).

## Results

Sample traits

Table 1 summarizes the primary sample traits, and Table 2 defines the study sample characteristics based on the Milan category. Testing against the hypothesis of uniform distribution by Chi-squared revealed that the sample’s Milan stage was not uniformly distributed (χ2 = 114.944 p < 0.001, φc = 0.334). A post hoc pairwise comparison indicated that stage IV-A was significantly more abundant (p ≤ 0.05) than any other stage, and all other stages could be considered of equal abundance. Of the binary traits (Gender, PMH, side, and Rx), %Rx+ significantly varied by Milan stage. Estimated marginal means showed that the only significant pairwise difference was between subjects at the most extreme Milan stage (VI) and Milan stage IV-A. We found no significant association between the Milan stage and any of the other sample traits.

**Table 1:** Sample characteristics *Quantities are counts (Milan stage, gender, side), mean ± SEM (age, height, weight, year), or percent “yes” (PMH, complications, smoking, Rx). PMH: Past medical history; Rx: Additional treatment; SEM: The standard error of the mean.

Traits	Quantization* N=172
Milan Stage I	12 (6.9%)
Milan Stage II	14 (8.1%)
Milan Stage III	6 (3.5%)
Milan Stage IV-A	119 (69.2%)
Milan Stage IV-B	7 (4.1%)
Milan Stage V	2 (1.2%)
Milan Stage VI	12 (6.9%)
Female gender	70 (40.7%)
Male gender	102 (59.3%)
PMH (yes)	48 (27.9%)
Left side	89 (51.7%)
Right side	83 (48.3%)
Rx (yes)	16 (9.3%)
Age (years)	43.0 ± 1.2
Height (cm)	164 ± 1
Weight (kg)	78.6 ± 1.4
Year	2020 ± 0
Complications (yes)	33 (19.2%)
Smoking (yes)	25 (14.5%)

**Table 2:** Sample characteristics by Milan category †Mean ± SEM PMH: Past medical history; Rx: Additional treatment; SEM: The standard error of the mean.

Milan	Count	Male	Female	PMX +	PMX -	Right	Left	Rx+	Rx−	Age (years)^†^	Height (m)	Weight (kg)
I	12	7	5	3	9	3	9	1	11	43.4 ± 5.0	1.6 ± 0.1	73.2 ± 7.4
II	14	7	7	4	10	5	9	2	12	42.4 ± 4.3	1.6 ± 0.0	79.3 ± 5.4
III	6	5	1	2	4	3	3	1	5	45.2 ± 10.0	1.7 ± 0.1	75.7 ± 8.6
IV-A	119	70	49	39	80	60	59	5	114	43.5 ± 1.4	1.7 ± 0.0	78.4 ± 1.7
IV-B	7	3	4	2	5	5	2	2	5	43.0 ± 6.7	1.6 ± 0.0	78.1 ± 3.9
V	2	2	0	1	1	0	2	1	1	53.0 ± 11.0	1.7 ± 0.0	68.0 ± 1.0
VI	12	8	4	11	1	7	5	7	5	36.7 ± 4.9	1.6 ± 0.0	89.3 ± 6.5

The risk of malignancy significantly differed by Milan category

Both marginal and conditional probabilities of non-neoplastic, benign, and malignant states were calculated. Marginal probabilities were dominated by the preponderance of Milan stage IV-A (69.2% of subjects). All told, malignancy was present in 12.7% of subjects. Overall marginal risk of malignancy by Milan category was (in descending order) 5.8% (VI), 2.9% (IV-A), 1.7% (I, III, and IV-B), 1.2% (II), and 0.6% (V). Conditional risks only estimate risk within (conditional upon) a specific Milan category (Table 3). Conditional probability by Milan category was (in descending order) 83.3% (VI), 50.0% (III and V), 42.9% (IV-B), 25.0% (I), 14.3% (II), and 4.2% (IV-A). Thus, both in an absolute sense and relative to its Milan stage, Milan category VI was associated with the highest risk of malignancy. This association was significant at p ≤ 0.05.

**Table 3:** Conditional probabilities of non-neoplastic, benign, or malignant by Milan category *Probability as percent ± SEM SEM: The standard error of the mean.

Milan	Non-neoplastic*	Benign	Malignant
I	25.0% ± 12.5%	50.0% ± 14.4%	25.0% ± 12.5%
II	64.3% ± 12.8%	21.4% ± 11.0%	14.3% ± 9.4%
III	0.0% ± 0.0%	50.0% ± 20.4%	50.0% ± 20.4%
IV-A	0.8% ± 0.8%	95.0% ± 2.0%	4.2% ± 1.8%
IV-B	0.0% ± 0.0%	57.1% ± 18.7%	42.9% ± 18.7%
V	0.0% ± 0.1%	50.0% ± 35.4%	50.0% ± 35.4%
VI	8.3% ± 8.0%	8.3% ± 8.0%	83.3% ± 10.8%

FNA had a moderate success rate overall

The total percentage of agreement was between 80.8% and 90.4% based only on Milan categories II, IV-A, and VI and the corresponding pathology classes. No pathological analyses were nondiagnostic, essentially creating an asymmetrical confusion matrix (Table 4). The Matthews correlation coefficient (MCC) indicated that as long as the FNA gave any diagnostic result, the overall quality of FNA versus pathology was 0.510−0.575.

**Table 4:** Confusion matrix for FNA vs. pathology FNA: Fine needle aspiration

		FNA			
		Malignancy	Benign	Non-neoplastic	Non-diagnostic
Pathology	Malignancy	11	9	4	3
	Benign	2	120	3	6
	Non-neoplastic	1	1	9	3
	Non-diagnostic	0	0	0	0

FNA's success was influenced by the Milan stage and Rx

According to the BIC, the most parsimonious model explaining FNA success/failure was Milan + Rx. Estimation of effects for each category indicated that the specific effects for Rx−, Rx+, and Milan I, II, Milan III, IV-A, and VI were reliable (p ≤ 0.05). However, within each Rx status, a pairwise comparison of rates of FNA success essentially showed that Milan stages IV-A and VI were associated with significantly higher success rates than Milan stages I and III, with all other stages being intermediate and not distinguishable from each other.

In our retrospective sample, FNA had an overall success rate of 81%, but MCC suggested a lower confidence overall (0.510, 0.575). Agreement between FNA and pathology was influenced by the Milan stage in a non-linear fashion and by the presence of additional Rx, where additional Rx was associated with lower predicted success. However, the sample size was restrictive. Three Milan categories had less than 10 members, and one had only two members. This work should be taken as suggestive and not conclusive. However, it does suggest that FNA, for all its convenience, is still unlikely to replace a more in-depth diagnostic method.

The sensitivity was 64% and specificity was 94% based on non-neoplastic masses. For benign masses, the sensitivity was 91% and specificity was 66%; however, the rates were 40% (sensitivity) and 97% (specificity) for malignant lesions (Table 5).

**Table 5:** Specific classification metrics

Specific Metrics	FNA Prediction			
	Malignant	Benign	Non-neoplastic	Nondiag
False discovery rate	0.214	0.077	0.438	1.000
False negative rate	0.593	0.084	0.357	NA
False omission rate	0.110	0.355	0.037	0.000
False positive rate	0.023	0.333	0.051	0.079
Positive likelihood ratio	17.926	2.748	12.673	NA
Negative likelihood ratio	0.606	0.126	0.376	NA
Negative predictive value	0.890	0.645	0.963	1.000
Positive predictive value	0.786	0.923	0.563	0.000
Sensitivity	0.407	0.916	0.643	NA
Specificity	0.977	0.667	0.949	0.921

## Discussion

The present study evaluated the accuracy of the MSRSGC for assessing the ROM and its correlation with post-surgical histopathological findings. Within our research, we noticed a balanced distribution between the left and right sides, indicating the absence of a notable preference for either side. This finding carries crucial implications, suggesting that the occurrence of these lesions is not inherently skewed toward any specific side. Furthermore, our paper demographically characterizes the study sample, revealing that males constituted 59.3% of the participants, in agreement with the published literature [7,10-12].

In terms of categorization, our findings revealed that the highest frequency of cases occurred within category IV-A (benign), followed by categories II (non-neoplastic) and VI (malignancy). These findings closely mirror outcomes by Pal et al. [13], who analyzed parotid lesions over three years, revealing a similar distribution with a higher prevalence of non-neoplastic (29.5%) and benign (51.3%) cases as compared to malignant tumors (19.2%). Correspondingly, Sheetal et al. [14], Yogambal et al. [15], and Karuna et al. [16] reported similar results, although their research covered salivary glands in a broader context rather than being exclusively confined to the parotid gland.

Furthermore, the current study calculated the ROM. Nguyen and Giang [12] and Reerds et al. [17] demonstrated similar ROM percentages for the parotid gland, as found in this study, which provides further substantiation for the consistency of ROM rates across MSRSGC categories. Nevertheless, these studies did exhibit slight deviations, specifically in relation to Milan category III (AUS). Our findings regarding this category align with the results of two independent studies. Rossi et al.’s [8] comprehensive analysis published in 2017 shed light on the anticipated ROM estimates for the distinct MSRSGC categories, with a ROM of 43% for cases classified as category III (AUS). Similarly, Johnson et al. [18] reported varying ROMs (ranging from 0% to 68%) for AUS across five diverse institutions.

Parallel to our findings, a similar multi-institutional study conducted on a distinct type of salivary gland tumor, submandibular gland lesions, revealed a parallel between the ROM values across various MSRSGC categories for submandibular gland FNA specimens and those previously documented for parotid gland FNA specimens [9]. This resonance in ROM values indicates a consistent applicability of the MSRSGC in diverse salivary gland tumor scenarios [9]. As evidenced by this example, the MSRSGC demonstrated its reliability not only in our parotid gland tumor study but also in the realm of submandibular gland lesions. Such coherence in ROMs emphasizes the utility of the MSRSGC in fostering improved patient management strategies, thereby underscoring its potential to be a valuable tool in guiding clinical decisions across varied salivary gland tumor types.

In line with existing literature [10-16], our study revealed an 80% agreement between the results of FNA across non-neoplastic, benign, and malignant instances and the final histopathological findings. Particularly in cases classified as malignant, our investigation showed a notable specificity rate of 98% for malignancies when FNA was utilized in conjunction with the Milan system, which falls within the reported range of 87-100% in the published literature [7,9-16,19,20]. However, our study’s sensitivity rate of 23% is low compared to the published literature (57.0-94.7%) [7,9-16,19,20]. This finding suggests an elevated risk of encountering false-negative results. This outcome may be attributed to the challenges posed by distinguishing between non-neoplastic tumors, benign neoplasms, and malignant tumors due to intratumoral cytomorphologic variations, metaplastic changes, and issues related to sampling [9,21,22]. The reduced sensitivity rates when identifying malignant lesions indicate that clinicians should exercise caution when solely depending on biopsy outcomes and should consider deferring to definitive surgical intervention.

The present study is subject to certain limitations, including its retrospective design. However, the present study was implemented within two tertiary healthcare centers. This multi-center approach strives to mitigate potential biases associated with a single-center study and offers insights that are more representative of diverse clinical settings.

## Conclusions

FNA stands as a safe, fast, and minimally invasive diagnostic modality. The employment of FNA in conjunction with the MSRSGC serves as a risk stratification framework, offering treatment guidance and facilitating communication between pathologists and surgeons. However, the limited sensitivity rates for diagnosing malignant lesions should alert clinicians not to rely overly on biopsy results and instead defer to definitive surgical management. In this context, it becomes evident that future investigations, including larger-scale studies, are essential to further elucidate and enhance the utility of this diagnostic tool.
